# *QuickStats:* Percentage of Adults Aged 50–75 Years Who Met Colorectal Cancer (CRC) Screening Recommendations*^,†^ — National Health Interview Survey, United States, 2018**^§^**

**DOI:** 10.15585/mmwr.mm6911a7

**Published:** 2020-03-20

**Figure:**
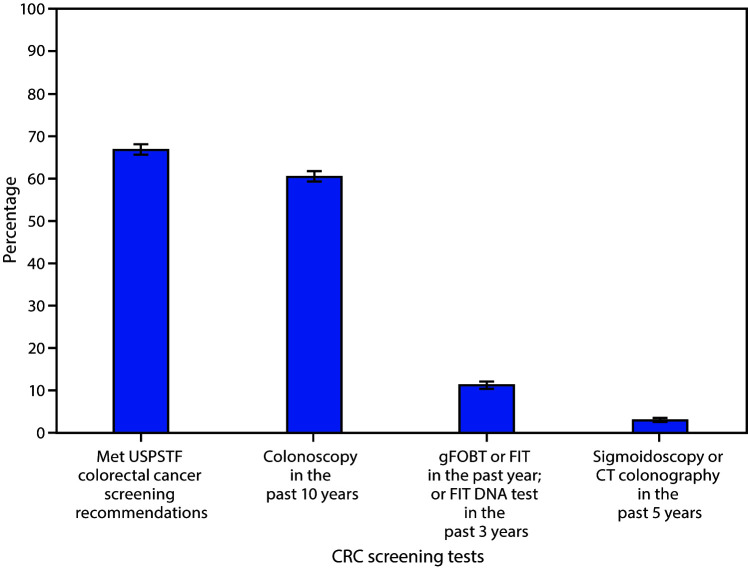
In 2018, 67.0% of U.S. adults aged 50–75 years met the U.S. Preventive Services Task Force recommendations for colorectal cancer screening; 60.6% had a colonoscopy in the past 10 years. An estimated 11.3% had either a gFOBT or FIT within the past 1 year, or had a FIT DNA test in the past 3 years. Fewer adults, 3.1%, had a sigmoidoscopy or CT colonography in the past 5 years.

**Source:** National Health Interview Survey, 2018. https://www.cdc.gov/nchs/nhis.htm.

